# Characterization of a Murine Model for *Encephalitozoon hellem* Infection after Dexamethasone Immunosuppression

**DOI:** 10.3390/microorganisms8121891

**Published:** 2020-11-29

**Authors:** Guozhen An, Yunlin Tang, Biying Mo, Maoshuang Ran, Xiao He, Jialing Bao, Zeyang Zhou

**Affiliations:** 1 State Key Laboratory of Silkworm Genome Biology, Southwest University, Chongqing 400715, China; agz19940913@email.swu.edu.cn (G.A.); t7anthony@email.swu.edu.cn (Y.T.); moowei1211@email.swu.edu.cn (B.M.); ranmshuang@email.swu.edu.cn (M.R.); zyzhou@swu.edu.cn (Z.Z.); 2 Chongqing Key Laboratory of Microsporidia Infection and Control, Southwest University, Chongqing 400715, China; 3 College of Sericulture, Textile and Biomass Sciences, Southwest University, Chongqing 400715, China; hx40097236@email.swu.edu.cn; 4 College of Life Sciences, Chongqing Normal University, Chongqing 401331, China

**Keywords:** microsporidia, *Encephalitozoon hellem*, dexamethasone, murine model, immunity

## Abstract

Background: *Encephalitozoon hellem* (*E. hellem*) belongs to a group of opportunistic pathogens called microsporidia. Microsporidia infection symptoms vary and include diarrhea, ocular disorders and systemic inflammations. Traditionally, immunodeficient animals were used to study microsporidia infection. To overcome the difficulties in maintenance and operation using immunodeficient mice, and to better mimic natural occurring microsporidia infection, this study aims to develop a pharmacologically immunosuppressed murine model of *E. hellem* infection. Methods: Wild-type C57BL/6 mice were immunosuppressed with dexamethasone (Dex) and then *E. hellem* spores were inoculated into the mice intraperitoneally. Control groups were the Dex-immunosuppressed but noninoculated mice, and the Dex-immunosuppressed then lipopolysaccharide (LPS)-treated mice. Mice body weights were monitored and all animals were sacrificed at the 15th day after inoculation. Tissue fragments and immune cells were collected and processed. Results: Histopathological analysis demonstrated that *E. hellem* inoculation resulted in a disseminated nonlethal infection. Interestingly, *E. hellem* infection desensitized the innate immunity of the host, as shown by cytokine expressions and dendritic cell maturation. We also found that *E. hellem* infection greatly altered the composition of host gut microbiota. Conclusions: Dex-immunosuppressed mice provide a useful tool for study microsporidiosis and the interactions between microsporidia and host immunity.

## 1. Introduction

Microsporidia are a group of intracellular pathogens causing opportunistic infections [1,2]. The most common species for mammals are *Encephalitozoon hellem* (*E. hellem*), *Encephalitozoon cuniculi* (*E. cuniculi*), *Enterocytozoon bieneusi* (*E. bieneusi*), and *Encephalitozoon intestinalis* (*E. intestinalis*) [3,4,5]. The infection outcomes and clinical symptoms are associated with pathogen species and hosts immune-state. Usually, in immunocompromised hosts the symptoms are more obvious including persistent diarrhea and even lethal outcomes [4,6,7]. In immunocompetent hosts the infections are usually asymptomatic, yet in immunocompetent rabbits, the clinical signs are shown after *E. cuniculi* infection [8,9,10]. So far, most of what is known about the host immune responses against microsporidia are from severe combined immunodeficiency (SCID) or nude animal models, using *E. cuniculi* or *E. intestinalis* as infecting pathogens [11,12,13]. Studies showed that cell-mediated immunity, especially the CD8+T lymphocytes mediated cytotoxic effect is important for hosts [14,15,16]. In comparison, the importance of host innate immunity against microsporidia has been less studied, yet has started to draw more attention recently [17,18]. Therefore, more studies about the interactions between microsporidia and innate immune components, with a broader range of microsporidia species as infecting agents are necessary to fully elucidate the interactions between microsporidia and host innate immunity.

Mice are one of the commonly used mammalian models to study host immunity against microsporidia [19]. In many cases, immunosuppression is necessary for a successful experimental infection. The immunosuppression could be achieved by either genetic modification or by drug administration. Genetically modified mice, such as SCID ones, usually require sophisticated manipulation and maintenance [20]. What’s more, in athymic or SCID mice, microsporidia infect various internal organs with probable lethal outcome [21]. Thus SCID mice were not an optimistic model to study microsporidia infection, for the chronic/latent infections in immunocompetent individuals are common yet generally asymptomatic [22].

In comparison, drug-immunosuppressed mice are much easier to manipulate and rear. Most importantly, microsporidia infections in these mice better mimic naturally occurring chronic/latent infections [23]. In most cases, the immunosuppression is attended by injection of glucocorticoids such as dexamethasone (Dex) [24,25]. The major changes of host immunity after Dex administration were fully characterized, reflected as lymphoid depletion and leukocytes inactivation [26,27]. As the results, Dex-induced immunosuppression may serve as a good approach to study the functions of host immunity, especially the innate immunity in response to pathogens invasion. Dex-induced immunosuppression has been applied in microsporidia–host interaction study, mostly with *E. cuniculi* and *E. intestinalis* as infecting agents; while *E. hellem* was relatively less studied [21,28,29,30].

Here in this study, we assessed the infection of *E. hellem* to Dex-immunosuppressed mice. Our study provides a murine model that mimics the naturally occurring latent infection of microsporidia and serves as a good platform to study the host innate immune responses against microsporidia.

## 2. Materials and Methods

### 2.1. Pathogen and Cell Cultures

*E. hellem* strain (ATCC 50504/50451) is gift of Prof. Louis Weiss (Albert Einstein College of Medicine, New York, NY, USA), and are reproduced in rabbit kidney cells (RK13, ATCC CCL-37). RK13 cells were cultured in 10% fetal bovine serum (FBS) (ThermoFisher, Waltham, MA, USA) containing Minimum Essential Medium Eagle (MEM) with penicillin-streptomycin at 5% CO_2_ until confluent and then infected with *E. hellem*. The spores were collected from culture media, purified by passing them through 5 μm size filter (Millipore, Billerica, MA, USA) to remove host cells, concentrated by centrifugation, and stored in sterile distilled water at 4 °C [31]. Spores used in these experiments were counted with a hemocytometer (three times/sample and averaged).

### 2.2. Animals and Experimental Design

Groups of C57BL/6 mice (six weeks in age, female) were reared in animal care facility according to the Southwest-University-approved animal protocol (SYXK-2017-0019). Dexamethasone-induced immnosuppression was administered to all mice, and achieved by intraperitoneal injection of dexamethasone (Aladin, Cas 2392-39-4, Shanghai, China) at the dose of 5 mg/kg/day for six days, method adopted from previous studies [26,28,32]. The experimental group of mice were then infected with 1 × 10^7^ *E. hellem* spores/day for two days; while the control groups of mice were then injected either with 1XPBS or lipopolysaccharide (LPS, 5 mg/kg). After these treatments, all mice were then housed for another 15 days and body weights were monitored, starting on the last day of dexamethasone-injection, then measured again on the day of mice sacrifice (end of experiment) for a total 18 days period of time. At the endpoint of the experiment, all mice were sacrificed by CO_2_ inhalation. Samples of blood, feces and various organs were collected from all mice for further investigations. *E. hellem*-infected mice and uninfected mice organs (colon, spleen and liver) were collected and frozen sectioned respectively. All sections were then stained by hematoxylin and eosin (H&E staining). Plasma samples were prepared from the peripheral blood of three groups of mice.

### 2.3. Detection of E. hellem Spores

Colonization of *E. hellem* spores within host organ tissues were detected by fluorescence staining with calcofluor white (CFW). The colonization of *E. hellem* in host were further confirmed by PCR assay to show the existence in immune cells, the dendritic cells, using primers 5′-TGAGAAGTAAGATGTTTAGCA-3′ and 5′-GTAAAAAGACTCTCACACTCA-3′ [33].

### 2.4. Cytokines Expressions

Peripheral blood samples were collected from mice of all three groups, the uninfected control, *E. hellem*-infected and LPS-treated. Sodium citrate was used as anticoagulant, and blood samples were centrifuged at 1000× *g* for 10 min to enrich plasma. All plasma samples were then subjected to cytokine expression assay, using mouse Interleukin 12 ELISA kit and Mouse IL-6 ELISA Kit (ThermoFisher Scientific, Waltham, MA, USA).

### 2.5. Dendritic Cells (DCs) Isolation

Spleens were collected from mice of all three groups, the uninfected control, *E. hellem*-infected and LPS treated. Isolation of DCs starts with mince the spleen into homogenous paste using commercially available Spleen Dissociation Medium (STEMCELL Technologies, Vancouver, Canada) in tissue culture dish, followed by gently passing several times through a 16 Gauge Blunt-End Needle attached to a 3 cc Syringe and then through a primed 70 μm nylon mesh filter. The filtered single cell suspension was then subjected to the EasySep Mouse Pan-DC Enrichment Kit (STEMCELL Technologies, Vancouver, Canada) to isolate dendritic cells only. This kit targets non-DCs for removal with biotinylated antibodies recognizing specific cell surface marker on unwanted cells in solution. Briefly, Enrichment Cocktail and Biotin Selection Cocktail, combinations of monoclonal antibodies, were added to the sample. Next, the samples were incubated with Streptavidin-bound magnetic particles to solution and a magnet was used to immunomagnetic negative selection of the dendritic cells. The purified DCs were then counted and cultured in RPMI Medium 1640 (supplemented with 10% FBS, 50 ng/mL GM-CFS (granulocyte-macrophage colony-stimulating factor) and penicillin/streptomycin) (Gibco, Waltham, MA, USA) in a 37 °C, 5% CO_2_ incubator.

### 2.6. Flow Cytometry Analysis

The isolated dendritic cells, either from uninfected control mice, *E. hellem* infected mice or LPS treated mice were all subjected to flow cytometer and analyzed for cell maturation. In brief, the DCs were firstly washed with 1× PBS/0.3% BSA, and then stained with FITC-conjugated anti-CD40 and PE-conjugated CD86 (BioLegend, San Diego, CA, USA) for 30 min at 4 °C. CD40+/CD86+ DCs were considered as more matured DCs. Flow cytometry analysis was performed by a FACSCanto II flow cytometer (BD Biosciences) and data were collected and analyzed with FACSDiva software (v6.1.2).

### 2.7. Gut Microbiota Analysis

The stools samples and colon contents were collected from all mice on the day of sacrifice, and the total DNAs were isolated respectively, using PowerSoil^®^ DNA Isolation kit. The total DNAs were then served as template to amplify the coding sequences for the 16S RNA (primers 5′- AGRGTTTGATYNTGGCTCAG-3′ and 5′- TASGGHTACCTTGTTASGACTT-3′); the 18S RNA (primers 5′- AACCTGGTTGATCCTGCCAGT-3′ and 5′- GATCCTTCTGCAGGTTCACCTAC-3′); and internal transcribed spacer (primers 5′- CTTGGTCATTTAGAGGAAGTAA-3′ and 5′- TCCTCCGCTTATTGATATGC-3′) [34]. PCR reactions were performed on an ABI GeneAmp 9700 PCR system (Applied Biosystems, Waltham, MA, USA). The amplicons were purified and trimmed barcodes, the remaining sequential reads of each sample were linked by FLASH V1.2.7 [35,36].

Taxonomy-based analyses were conducted using the Ribosomal Database Project Naive Bayes classifier, with a confidence of 95%. The remaining sequences with more than 97% similarity threshold were clustered into OTUs [37]. Each read was classified to phylum, class, order, family, and genus with the SILVA reference database. The relative abundance of species was calculated by dividing the number of sequences of each taxonomic level by the total number of sequences per sample [38].Microbiome diversity was estimated by alpha diversity (within communities) and beta diversity (between microbial communities) analyses. Alpha diversity evaluated species richness with ACE index and Chao 1 index, diversity with Shannon and Simpson indexes [39].

### 2.8. Statistical Analysis

Each experiment includes various numbers of samples (n), and each experiment has been repeated at least three times. Statistical analysis of results was conducted by using one-way ANOVA and Student’s *t*-test to identify the differences between two groups, with *p* < 0.05 being considered a significant difference.

## 3. Results

### 3.1. Subsection

#### 3.1.1. Colonization of E. hellem in Dexamethasone-Immunosuppressed Mice

**Figure 1:**
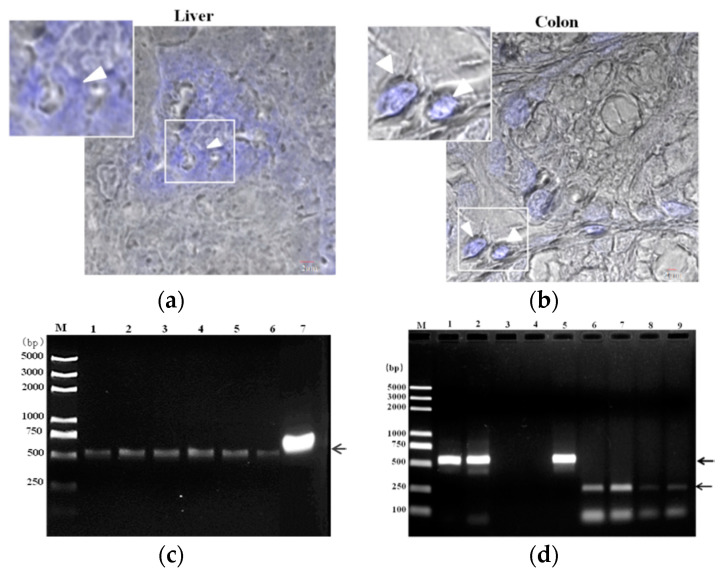
Colonization of *E. hellem*. (**a**) Liver and (**b**) colon samples were collected from *E. hellem* infected mice, immediately frozen by liquid nitrogen, and then prepared by frozen sectioning. The existences of *E. hellem* were detected by calcofluor white (CFW) staining, as shown in blue color (also pointed out by white arrow heads) (scale bar = 2 μm). (**c**) Dendritic cells (DCs) isolated from *E. hellem* infected mice spleen, the cDNAs were then prepared. A representative image of PCR results using six individual DCs-cDNA samples as template (Lane 1–6), and *E. hellem* total DNA as positive template control (Lane 7) is shown. All samples showed positive results (as pointed out by arrow), indicating the existence of *E. hellem* in host DCs. (**d**) Lane 1 and 2 are representative two DCs-cDNA samples from *E. hellem* infected mice as template with *E. hellem* specific primers (arrow shows the positive band at 547 bp); Lane 3 and 4 are representative two DCs-cDNA samples from un-infected mice as template with *E. hellem* specific primers; Lane 5 is *E. hellem* total DNA as positive template control (arrow shows the positive band at 547 bp); Lane 6 and 7 are the same two DCs-cDNA samples as in Lane 1 and 2 respectively, and are used as templates with actin specific primers (arrow shows the positive band at 250 bp); Lane 8 and 9 are the same two DCs-cDNA samples from lane 3 and 4 respectively, and are used as templates with actin specific primers (arrow shows the positive band at 250 bp).

Histological examination of mouse organs, such as liver and colon showed colonization of *E. hellem* spores (Figure 1), detected by CFW staining (Figure 1a). The following examination of isolated dendritic cells, by PCR using *E. hellem* specific primers, also confirmed the successful colonization of *E. hellem* within host body and immune cells (Figure 1b). Together, these results showed that dexamethasone-immunosuppression facilitates the successful infection and colonization of *E. hellem* within the host.

#### 3.1.2. Effects of *E. hellem* Infection on Body Weight

**Figure 2:**
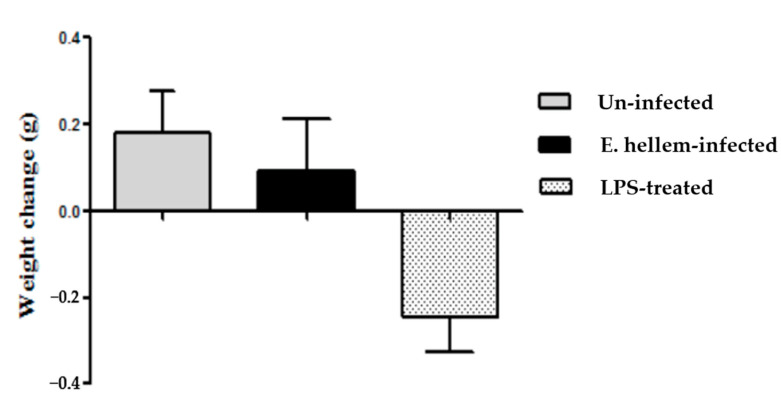
Body weights change. Body weights of all three groups of mice (uninfected, *E. hellem*-infected, and lipopolysaccharide (LPS)-treated) were measured. The first measurements were done on the last day of dexamethasone-injection and then measured again on the day of mice sacrifice, for a total 18 days period of time. The body weight changes over time were calculate and are represented in the figure. LPS-treated mice lost about 0.2 g of body weight over time, while uninfected mice and *E. hellem*-infected mice gain about 0.2 g of body weight at the end of the experiment. No significant differences among groups were observed. (*n* = 8 for each group in one experiment, and the experiments have been repeated for three times).

The body weights of all three groups of mice were monitored. Compared to the LPS-treated mice, the uninfected control and *E. hellem* infected mice did not lose any weight (Figure 2).

#### 3.1.3. Histopathological Analysis of *E. hellem*-Infected Mice

**Figure 3:**
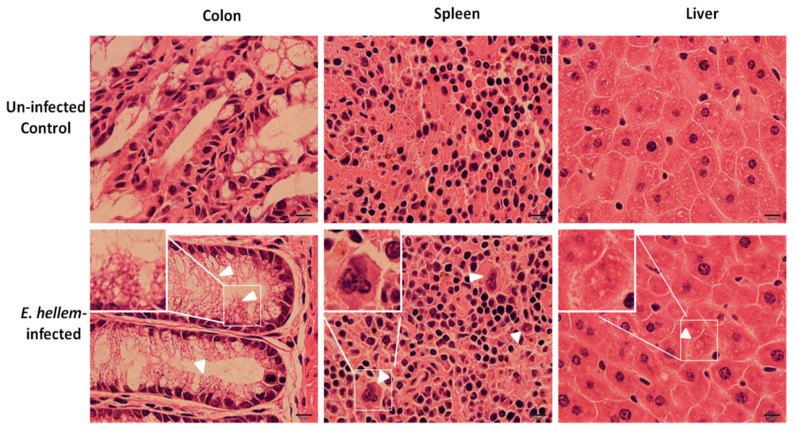
Histopathological analysis. Uninfected control and *E. hellem*-infected mice organs (colon, spleen and liver) were collected and frozen sectioned respectively. All sections were then subjected to hematoxylin and eosin staining. Here are the representative images of few *E. hellem*-infected organ samples with histopathological changes. In colon, enlarged microvilli were observed (white arrow heads); In spleen and liver, lymphoepithelioid granulomas were seen (white arrow heads). Images are representatives of all samples from eight control and infected mice respectively. (scale bar = 10 μm).

Consistently with our previous results, no significant signs of inflammation or organ damages were observed in the *E. hellem*-infected group. Yet in few cases, around three in all eight *E. hellem*-infected mice, histopathological changes were seen (Figure 3). In the colon, enlarged microvilli were observed, indicating swelling or edema caused by local inflammation. In spleen and liver, lymphoepithelioid granulomas were seen showing signs of interstitial nephritis or infiltrating lymphocytes.

#### 3.1.4. Cytokines Expressions Affected by *E. hellem* Infection

**Figure 4:**
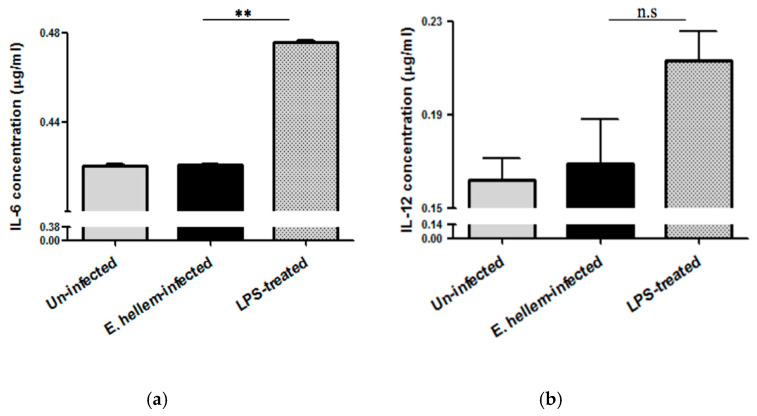
Cytokines expressions. Plasma samples were prepared from the peripheral blood of uninfected, *E. hellem*-infected and LPS-treated mice respectively. (**a**) Cytokine IL-6 expression levels were measured by mouse IL-6 ELISA Kit. Results showed that LPS would significantly upregulate IL-6 expression level, while *E. hellem* infection did not upregulate IL-6 expression (** = *p* < 0.01, *n* = 8 for each group). (**b**) Il-12 expression levels were quantified by mouse IL-12 ELISA Kit. Results also confirmed that *E. hellem* infection did not upregulate IL-12 expression, although no statistical differences among groups (n.s = No significance, *n* = 8 for each group in one experiment, and the experiments have been repeated for three times).

Inflammatory cytokines IL-6 and Il-12 expression levels were quantified using specific ELISA kits. The results showed that *E. hellem*-infection did not upregulate either IL-6 or IL-12 expression, as shown in Figure 4. These findings further demonstrate that our model mimics latent microsporidia infection which is consistent with our previous results.

#### 3.1.5. Dendritic Cells Maturation Affected by *E. hellem* Infection

**Figure 5:**
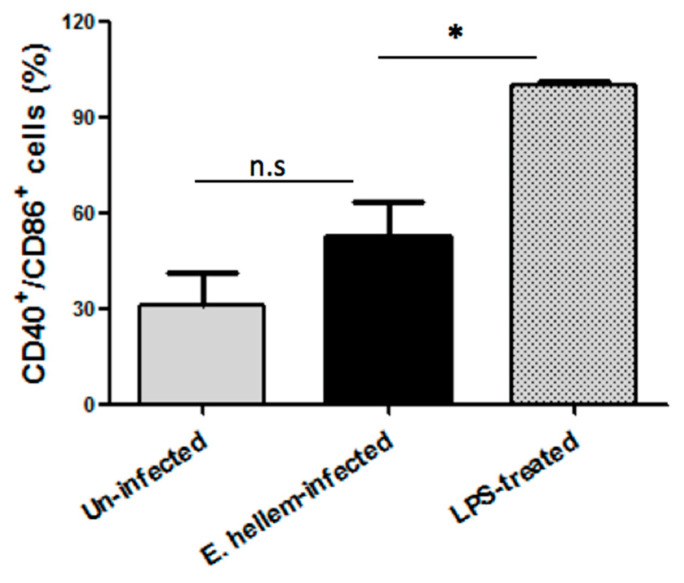
Flow cytometry analysis of DCs maturation. DCs isolated from uninfected control mice, *E. hellem*-infected mice and LPS-treated mice were subjected to flow cytometry analysis. The amounts of CD40^+^/CD86^+^ double positive DCs were recorded, and the ratios of uninfected to LPS-treated group and *E. hellem*-infected to LPS-treated group were calculated and presented in figure. As shown, DCs from either *E. hellem*-infected or uninfected group have significant lower numbers of matured cells. (* = *p* < 0.05, *n* = 8 for each group in one experiment, and the experiments have repeated for three times).

The matured DCs were determined by flow cytometry using fluorescent labeled anti-CD40 and anti-CD86 antibodies, and LPS-treated group were assigned as positive control. The amounts of CD40+/CD86+ double positive DCs were recorded and the ratios were calculated. As shown in Figure 5, *E. hellem* infection did not promote DCs maturation as compared to LPS treatment. This result again confirmed our previous data, that our model mimics latent microsporidia infection and this kind of infection is more likely to happen in nature.

#### 3.1.6. Mice Gut Microbiota Affected by *E. hellem* Infection

**Figure 6:**
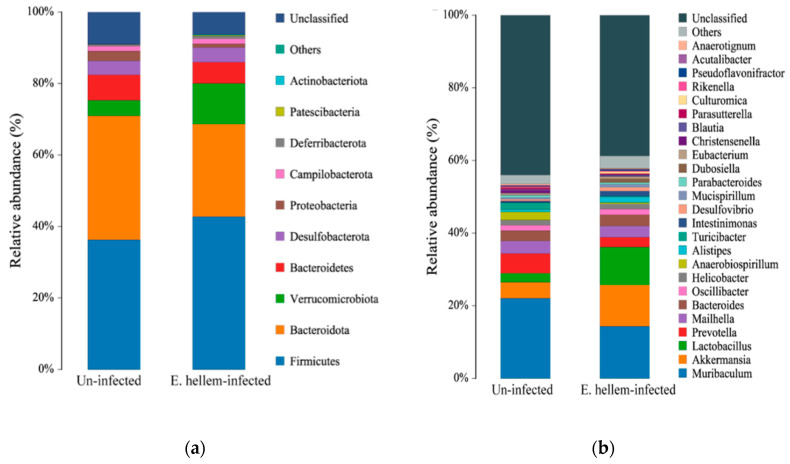
Composition of the gut bacteria in *E. hellem*-infected and uninfected group. The relative abundance of phylum-level (**a**) and genus-level (**b**) gut bacteria taxa were analyzed and showed in figure. The results showed *E. hellem* infection greatly changed the microbiota composition, both on phylum and genus levels.

Relative abundance of microbiota of each group was compared on phylum and genus level, as shown in Figure 6. Our findings are as follows: although Shannon index showed no significant differences between the *E. hellem*-infected and uninfected groups; the microorganisms’ composition, especially the bacteria changed a lot after *E. hellem* infection. It’s shown in the figure that the abundance of Firmicutes and Verrucomicrobiota phylum turn to be more abundant after *E. hellem* infection. The abundances of Bacteroidota and Bacteroidetes phylum decrease after *E. hellem* infection. On the genus level, *Akkermansia* and *Lactobacillus* rise after *E. hellem* infection, while *Turicibacter* and *Parasutterella* decrease after *E. hellem* infection. Together, *E. hellem* infection greatly disturbs the host microbiota composition.

## 4. Discussion

In this study, we established and evaluated a murine model of microsporidia infection. *E. hellem* is the one of the most commonly found human-infecting microsporidia pathogens but relatively less studied on murine models [4]. Here in this study, we firstly administered dexamethasone into wild-type mice to facilitate microsporidia invasion, then *E. hellem* was inoculated and successfully colonized within various organ and immune cells. These results showed that dexamethasone treatment is suitable in establishing the murine model of *E. hellem* infection. Our results also showed that *E. hellem* infection is systemic yet nonlethal, and evokes limited levels of host immune response. We also found that *E. hellem* infection altered host gut microbiota. These results confirmed that *E. hellem* infection of the dexamethasone-immunosuppressed mice mimics the natural occurring or latent microsporidia infection, and provides a valuable murine model for future studies.

Traditionally, microsporidia is considered as opportunistic pathogens and microsporidia infects immunodeficient hosts [40,41]. However, accumulating evidence pointed out the potential of microsporidia infecting host naturally and even in immune-competent individuals [7,22]. These infections could be latent or symptomatic depends on the species and virulence of pathogens [9,10]. The ‘living-together’ strategy reflects a balanced pathogen-host relationship [42]. This inference is supported by our findings that *E. hellem* could successfully colonize within host but could not evoke significant organ damage or systemic inflammation. We believe current study mimics the latent infection model of *E. hellem*. It is interesting to notice the fact that latent microsporidiosis could develop to reactivation and redissemination [21,43]. Our study proves this inference in a certain way, as we showed that *E. hellem* infection may desensitize the host immunity, leads to less proinflammatory cytokines expressions and delayed DCs maturation. We also investigated other functionalities of DCs after *E. hellem* infection, including the surface marker expressions and signaling pathway changes (data now shown). Dexamethasone is a widely used anti-inflammatory drug, and may also alter the host gut microbiota composition and facilitate opportunist infections [44,45]. We used dexamethasone only to facilitate the successful infection or colonization of *E. hellem* in mice. As a matter of fact, we designed our study so that all mice, including the ‘*E. hellem*-infected’, ‘uninfected’ and ‘LPS-treated’ mice, were all dexamethasone pretreated. In this way, we could exclude the effects of dexamethasone on host immune state, and only compare the differences before and after *E. hellem* infection and to better investigate the roles of *E. hellem* on the host.

It has been reported that microsporidia infection of immunocompetent humans may cause a short acute diarrheal phase followed by asymptomatic infection, or chronic malabsorbtive diarrhea may develop [46]. In our study we found that *E. hellem* infection altered host gut microbiota, and these findings may help to explain the above phenomena. We found that after *E. hellem* infection, the relative abundances of Firmicutes and Bacteroidetes phyla changed significantly with the former increase while the later decrease. It is known that the intestinal microbiota consists of over 1000 various bacterial species, mainly belonging to Firmicutes and Bacteroidetes phyla, containing both beneficial and pathogenic microbes [47]. Thus the changes of these two phyla after *E. hellem* infection would significantly modify the host metabolism and immunity. In addition, we found that the relative abundance of Blautia phylum increased after *E. hellem* infection. A recent study pointed out the association between Blautia and Crohn’s disease [48]. These findings may help to explain the acute or persistent diarrheal and malabosorbtion in microsporidia infected individuals. Our findings on genus levels also revealed multiple changed groups and the results are worthy of further investigation. For example, we found the relative abundance of genus *Akkermansia* increase after *E. hellem* infection. *Akkermansia muciniphila* is a rising star in the control of obesity and diabetes [49], thus it will be reasonable to infer that *E. hellem* infection altered the metabolism of host. In addition, the abundances of genus *Parasutterella* and *Turicibacter* were downregulated. The former is found to be core component of gut microbiota, and later one is important for host to make serotonin, an important neurotransmitter and chemical to reduce inflammation [50,51]. *E. hellem* infection caused decrease of these two components, may help to explain the dysbiosis of host microbiota and the desensitized immunity.

## 5. Conclusions

In conclusion, dexamethasone-induced immunosuppression facilitates *E. hellem* infection on wild-type mice. This murine infection model better mimics the naturally occurring and latent microsporidia infection. Our findings showed that this kind of infection is mostly asymptomatic yet *E. hellem* can successfully reproduce within the host body. We further demonstrated that *E. hellem* infection may desensitize host immunity and disturbed host gut microbiota. Together, our model provides a great tool to study microsporidia and host interactions.

## References

[1] Pan G., Bao J., Ma Z., Song Y., Han B., Ran M., Li C., Zhou Z. (2018). Invertebrate host responses to microsporidia infections. Dev. Comp. Immunol..

[2] Han B., Weiss L.M. (2017). Microsporidia: Obligate Intracellular Pathogens within the Fungal Kingdom. Microbiol. Spectr..

[3] del Aguila C., Lopez-Velez R., Fenoy S., Turrientes C., Cobo J., Navajas R., Visvesvara G.S., Croppo G.P., Da Silva A.J., Pieniazek N.J. (1997). Identification of Enterocytozoon bieneusi spores in respiratory samples from an AIDS patient with a 2-year history of intestinal microsporidiosis. J. Clin. Microbiol..

[4] Franzen C., Müller A. (2001). Microsporidiosis: Human diseases and diagnosis. Microbes Infect..

[5] Mathis A., Weber R., Deplazes P. (2005). Zoonotic Potential of the Microsporidia. Clin. Microbiol. Rev..

[6] Cisláková L., Halanova M. (2003). [Microsporidial infections in immunocompromised hospitalized patients]. Epidemiol. Mikrobiol. Imunol. Cas. Spol. Epidemiol. Mikrobiol. Ceske Lek. Spol. JE Purkyne.

[7] Weber R., Bryan R.T. (1994). Microsporidial infections in immunodeficient and immunocompetent patients. Clin. Infect. Dis. Off. Publ. Infect. Dis. Soc. Am..

[8] Didier E.S., Weiss L.M. (2011). Microsporidiosis: Not just in AIDS patients. Curr. Opin. Infect. Dis..

[9] Schmidt E.C., Shadduck J.A. (1983). Murine encephalitozoonosis model for studying the host-parasite relationship of a chronic infection. Infect. Immun..

[10] Künzel F., Fisher P.G. (2018). Clinical Signs, Diagnosis, and Treatment of Encephalitozoon cuniculi Infection in Rabbits. Veter Clin. N. Am. Exot. Anim. Pract..

[11] Khan I.A., Moretto M., Weiss L.M. (2001). Immune response to Encephalitozoon cuniculi infection. Microbes Infect..

[12] Salat J., Sak B., Le T., Kopecký J. (2004). Susceptibility of IFN-gamma or IL-12 knock-out and SCID mice to infection with two microsporidian species, Encephalitozoon cuniculi and E. intestinalis. Folia Parasitol..

[13] Kotková M., Sak B., Hlásková L., Květoňová D., Kváč M. (2018). Evidence of transplacental transmission of Encephalitozoon cuniculi genotype II in murine model. Exp. Parasitol..

[14] Braunfuchsová P., Salát J., Kopecký J. (2001). CD8+ T lymphocytes protect SCID mice against Encephalitozoon cuniculi infection. Int. J. Parasitol..

[15] Khan I.A., Schwartzman J.D., Kasper L.H., Moretto M. (1999). CD8+ CTLs are essential for protective immunity against Encephalitozoon cuniculi infection. J. Immunol..

[16] Salát J., Braunfuchsová P., Kopecký J., Ditrich O. (2002). Role of CD4+ and CD8+ T lymphocytes in the protection of mice against Encephalitozoon intestinalis infection. Parasitol. Res..

[17] Mathews A., Hotard A., Hale-Donze H., Hotard A.L. (2009). Innate immune responses to Encephalitozoon species infections. Microbes Infect..

[18] Bernal C.E., Zorro M.M., Esierra J., Egilchrist K., Botero-Garcés J.H., Ebaena A., Ramirez-Pineda J.R. (2016). Encephalitozoon intestinalis Inhibits Dendritic Cell Differentiation through an IL-6-Dependent Mechanism. Front. Cell. Infect. Microbiol..

[19] Tao L., Reese T.A. (2017). Making Mouse Models That Reflect Human Immune Responses. Trends Immunol..

[20] Nonoyama S., Ochs H.D. (1996). Immune Deficiency in SCID Mice. Int. Rev. Immunol..

[21] Kotkova M., Sak B., Kvetonova D., Kvac M. (2013). Latent Microsporidiosis Caused by Encephalitozoon cuniculi in Immunocompetent Hosts: A Murine Model Demonstrating the Ineffectiveness of the Immune System and Treatment with Albendazole. PLoS ONE.

[22] Sak B., Kváč M., Kučerová Z., Květoňová D., Saková K. (2011). Latent Microsporidial Infection in Immunocompetent Individuals—A Longitudinal Study. PLoS Negl. Trop. Dis..

[23] Lallo M.A., dos Santos M.J., Bondan E.F. (2002). [Experimental Encephalitozoon cuniculi infection in dexamethasone-immunosuppressed mice]. Rev. Saude Publica.

[24] Claman H.N. (1972). Corticosteroids and Lymphoid Cells. N. Engl. J. Med..

[25] Weston W.L., Mandel M.J., Krueger G.G., Claman H.N. (1972). Differential Suppressive Effect of Hydrocortisone on Lymphocytes and Mononuclear Macrophages in Delayed Hypersensitivity of Guinea Pigs. J. Investig. Dermatol..

[26] Jeklova E., Leva L., Jaglic Z., Faldyna M. (2008). Dexamethasone-induced immunosuppression: A rabbit model. Veter Immunol. Immunopathol..

[27] Schwarz E., Saalmuller A., Gerner W., Claus R. (2005). Intraepithelial but not lamina propria lymphocytes in the porcine gut are affected by dexamethasone treatment. Veter Immunol. Immunopathol..

[28] Herich R., Levkutova M., Kokinčáková T., Reiterovã K., Hipíková V., Levkut M. (2006). Diagnosis and Manifestation of Encephalitozoonosis in Mice after Experimental Infection with Different Species and Application of Dexamethasone. J. Veter Med. Ser. A.

[29] Nevárez-Garza A.M., Castillo-Velázquez U., Soto-Domínguez A., Montes-De-Oca-Luna R., Zamora-Ávila D.E., Wong-González A., Rodríguez-Tovar L.E. (2018). Quantitative analysis of TNF-α, IL-4, and IL-10 expression, nitric oxide response, and apoptosis in Encephalitozoon cuniculi—Infected rabbits. Dev. Comp. Immunol..

[30] Rodriguez-Tovar L.E., Castillo-Velazquez U., Arce-Mendoza A.Y., Nevarez-Garza A.M., Zarate-Ramos J.J., Hernandez-Vidal G. (2016). Interferon gamma and interleukin 10 responses in immunocompetent and immunosuppressed New Zealand White rabbits naturally infected with Encephalitozoon cuniculi. Dev. Comp. Immunol..

[31] Visvesvara G.S., Leitch G.J., Moura H., Wallace S., Weber R., Bryan R.T. (1991). Culture, electron microscopy, and immunoblot studies on a microsporidian parasite isolated from the urine of a patient with AIDS. J. Protozool..

[32] Venugopalan S.K., Shanmugarajan T.S., Navaratnam V., Mansor S.M., Ramanathan S. (2016). Dexamethasone provoked mitochondrial perturbations in thymus: Possible role of N-acetylglucosamine in restoration of mitochondrial function. Biomed. Pharmacother..

[33] Weiss L.M., Vossbrinck C.R. (1998). Microsporidiosis: Molecular and Diagnostic Aspects. Math. Model. Negl. Trop. Dis. Essent. Tools Control. Élimin. Part B.

[34] Sundberg C., Al-Soud W.A., Larsson M., Alm E., Yekta S.S., Svensson B.H., Sørensen S.J., Karlsson A. (2013). 454 pyrosequencing analyses of bacterial and archaeal richness in 21 full-scale biogas digesters. FEMS Microbiol. Ecol..

[35] Kõljalg U., Nilsson R.H., Abarenkov K., Tedersoo L., Taylor A.F.S., Bahram M., Bates S.T., Bruns T.D., Bengtsson-Palme J., Callaghan T.M. (2013). Towards a unified paradigm for sequence-based identification of fungi. Mol. Ecol..

[36] Edgar R.C. (2013). UPARSE: Highly accurate OTU sequences from microbial amplicon reads. Nat. Methods.

[37] Cole J.R. (2004). The Ribosomal Database Project (RDP-II): Sequences and tools for high-throughput rRNA analysis. Nucleic Acids Res..

[38] Quast C., Pruesse E., Yilmaz P., Gerken J., Schweer T., Yarza P., Peplies J., Glockner F.O. (2013). The SILVA ribosomal RNA gene database project: Improved data processing and web-based tools. Nucleic Acids Res..

[39] Robertson C.E., Harris J.K., Wagner B.D., Granger D., Browne K., Tatem B., Feazel L.M., Park K., Pace N.R., Frank D.N. (2013). Explicet: Graphical user interface software for metadata-driven management, analysis and visualization of microbiome data. Bioinformatics.

[40] Cavert W. (1997). Preventing and treating major opportunistic infections in AIDS. What’s new and what’s still true. Postgrad. Med..

[41] Weiss L.M. (1995). … and now microsporidiosis. Ann. Intern. Med..

[42] Vavra J., Lukes J. (2013). Microsporidia and ‘the art of living together’. Adv. Parasitol..

[43] Cali A., Kotler D.P., Orenstein J.M. (1993). Septata Intestinalis, N. G., N. Sp., an Intestinal Microsporidian Associated with Chronic Diarrhea and Dissemination in Aids Patients. J. Eukaryot. Microbiol..

[44] Zhao H., Jiang X., Chu W. (2020). Shifts in the gut microbiota of mice in response to dexamethasone administration. Int. Microbiol..

[45] Ünsal H., Balkaya M., Ünsal C., Biyik H., Basbulbul G., Poyrazoğlu E., Bıyık H. (2007). The Short-Term Effects of Different Doses of Dexamethasone on the Numbers of some Bacteria in the Ileum. Dig. Dis. Sci..

[46] Waywa D., Kongkriengdaj S., Chaidatch S., Tiengrim S., Kowadisaiburana B., Chaikachonpat S., Suwanagool S., Chaiprasert A., Curry A., Bailey W. (2001). Protozoan enteric infection in AIDS related diarrhea in Thailand. Southeast Asian J. Trop. Med. Public Health.

[47] Koliarakis I., Messaritakis I., Nikolouzakis T.K., Hamilos G., Souglakos J., Tsiaoussis J. (2019). Oral Bacteria and Intestinal Dysbiosis in Colorectal Cancer. Int. J. Mol. Sci..

[48] Schirmer M., Garner A., Vlakamis H., Xavier R.J. (2019). Microbial genes and pathways in inflammatory bowel disease. Nat. Rev. Genet..

[49] Everard A., Belzer C., Geurts L., Ouwerkerk J.P., Druart C., Bindels L.B., Guiot Y., Derrien M., Muccioli G.G., Delzenne N.M. (2013). Cross-talk between Akkermansia muciniphila and intestinal epithelium controls diet-induced obesity. Proc. Natl. Acad. Sci. USA.

[50] Fung T.C., Vuong H.E., Luna C.D.G., Pronovost G.N., Aleksandrova A.A., Riley N.G., Vavilina A., McGinn J., Rendon T., Forrest L.R. (2019). Intestinal serotonin and fluoxetine exposure modulate bacterial colonization in the gut. Nat. Microbiol..

[51] Ju T., Kong J.Y., Stothard P., Willing B.P. (2019). Defining the role of Parasutterella, a previously uncharacterized member of the core gut microbiota. ISME J..

